# Dr. Eleazor Price

**Published:** 1902-03

**Authors:** 


Dr. Eleazor Price, U. of B. '55, died at his home in Jackson, Mich., January 18, 1902. His age was about 70 years.

